# Annus Medicus: 1893

**Published:** 1894-01-06

**Authors:** 


### The Hospital

An Institutional Journal of

The Medical Sciences and Hospital Administration, with

Vol. XV.—No. 380.] AN EXTRA NURSING SUPPLEMENT. [Jan. 6, 1894.

### Annus Medicus: 1893.

At the close of the year 1893 the medical chronicler is able to congratulate his readers and himself upon the stimulating fact that medicine, both as a science and an art, is progressive, fresh, and full of vitality. In an age, energetic and eager beyond all parallel for practical advancement, medicine has attained a position of undoubted leadership: it heads an intellectual movement. As in the sixteenth century men looked to Luther, Erasmus, and the other reformers of religion, and as in more recent years they fixed their eyes upon the revolutionary movements of revolutionary France, so they now look, some with hope and some with apprehension, upon the newest discoveries in the biologico-medical sciences, and upon the changes of mental attitude in contemporary leaders of thought which spring therefrom. The average doctor, who fifty years ago was of no intellectual account, is now in very many instances the most intellectual and the most interesting man of his circle, or even of his neighbourhood.

### Bacteriology.

There are two lines of progress upon which the medical chronicler must constantly fix his eyes—the progress of medical science, and the progress of the medical art. We must surely also, in estimating the year's advance in medicine, bear in mind two things: first, that the year 1893 contained, like other years, twelve months only; and, secondly, that we have arrived at such an elevation of knowledge in the medical sciences and arts that progress is inevitably slow, and must become slower and slower as year by year advances. This is not written by way of apology for work not attempted and not done. Never, indeed, was more work attempted. The *Journal of Pathology* already referred to, and which has recently completed the first year of its active life, is a record charged with the most admirable evidences of strenuous work from most of the leading research laboratories in both hemispheres. Merely to mention the names of some of the workers and the nature of the researches in which they have been engaged is to show to all who concern themselves with the subject what a host of competent scientific enthusiasts are either doing pioneer work or going more thoroughly over ground that has been gone over before with the object of correction, confirmation, or fuller and more comprehensive interpretation. For example, in bacteriology Lockhart Gillespie has discussed the "Bacteria of the Stomach," C. S. Sherrington has published "Experiments on the Escape of Bacteria with the Secretions," and Butler Harris has contributed an original paper on a "New Micro-organism of Spreading Œdema." These are but the merest samples of the work which is everywhere being carried on in the bacteriological regions of this increasingly wonderful world.

### Phagocytosis and Tuberculosis.

One of the most interesting results of modern physiological research is the now practically established doctrine of phagocytosis; and perhaps the most important of all the speculations which have arisen out of this doctrine is the conclusion of Metchnikoff that the giant cells with which all medical men are familiar in connection with tuberculosis are really gigantic phagocytes. It will be remembered that four or five years ago Metchnikoff made some experiments on animals which are known to be specially resistent to tuberculosis. It is obvious that if an animal be specially resistent to tuberculosis, or indeed to any other disease, there must be a special reason for its special resistance. It is for science to find out that reason. *Spermophilus*, a small rodent which is found in Russia, is an example of an animal which has been found to be specially resistent to tuberculosis. Spermophilus, when inoculated with the tubercle bacillus, does indeed develop large glandular tumours of a tuberculous character. But there the tubercular process seems to become abortive, and the animal generally recovers by its own inherent power of self-recovery. Why is this? Metchnikoff, experimenting upon this little rodent, discovered that in the glandular tumours which resulted from his inoculation giant cells were found, and within the giant cells, deprived apparently of their power of destruction, were tubercle bacilli. Metchnikoff carefully observed the various phenomena which accompanied the process of bacilliary destruction. The bacilli gradually lost their power of reacting to staining agents, they swelled up, they became surrounded with several layers of yellowish structureless material, and finally lost all resemblance to ordinary bacilli. In another little rodent—*Meriones*—the bacilli within the giant cells seemed to become transformed into peculiar laminated bodies which, taking up lime salts, ultimately came to resemble the chalky concretions found in the lungs of tuberculous human subjects. This conclusion propounded by Metchnikoff has been extensively disputed, but Knud Faber undertook a series of experiments which he published last year, the results of which he holds are such as to practically substantiate Metchnikoff's conclusions. To use his own words—"I have succeeded in observing one additional process in which the giant cells in the most convincing manner possible appeared as intracellular digesting phagocytes." Manifold are the vistas opened out by these interesting researches and deductions, and the theme is most tempting if space did not rigorously forbid.

### Parasitic Protozoa in Cancer.

The other *bête noire* of medicine, cancer, has engaged the attention of some of the most distinguished and competent investigators both in our own and other countries. In June M. Armand Ruffer and H. G. Plimmer published a continuation of their investigations, entitled "Further Researches on some Parasitic Protozoa found in Cancerous Tumours." The observations made by these gentlemen were minute, painstaking, philosophical, and in every way worthy of the subject; and their photographic reproductions were beautiful and instructive in the highest degree. In October a further contribution, with still more beautiful coloured drawings, enhanced the interest of the discussion. Whether these parasitic protozoa, described by Ruffer and Plimmer, will ultimately be found to be the actual *fons et origo* of cancer or not we do not care at present to hazard a speculation. On this point Messrs. Ruffer and Plimmer conclude their article thus: "We have purposely abstained from entering into the question of the parasites as etiological factors, reserving this part of our subject until we have finished the examination of the cancer material at our disposal, from both man and animals." By way of criticism and comment on this subject may be mentioned the conclusions arrived at by Dr. Lindsay Steven and Mr. John Brown, who jointly undertook a special research on the subject of the parasitic protozoa of cancer. These gentlemen proceeded with great caution and competent skill. If the subject were not so serious one would like to smile at the differences between their paper and that of Messrs. Ruffer and Plimmer, and at the very considerable variations even in the plain matters of fact which the microscope seems to have revealed to the two sets of observers. The final conclusions arrived at by Messrs. Lindsay Steven and John Brown are expressed in a single sentence: "In conclusion," say they, "we have to say that we can go no further in the expression of an opinion as to the nature of the inclusion bodies" (the so-called parasitic protozoa of cancer) "than to state that they seem to us to be organised elements."

### The Therapeutics of the Year.

What of the new therapeutics of the year? The working doctor, it must be remembered, is a practical being, and his patients are, if anything, more practical still. We cannot expect them to be interested in cancer coccidia, or the phagocytic proclivities of giant cells, still less in such subjects as the "Intrinsic Innervation of the Kidney," the study of which has so profitably employed the leisure of Mr. Henry J. Berkley during the year just past. The "merely practical" man is not to be derided: he is to be helped. It is he who fights the actual battles of medicine, whilst the "professor of tactics" pursues his more fascinating studies in the laboratory. To confess the truth at once, there have been no startling advances made in the therapeutic art during the year 1893. And for our part we do not regret this. Startling advances in nine cases out of ten turn out to be startling failures. What we can congratulate ourselves upon is the undeniable fact of a steady advance of the therapeutic army along the whole line, in the direction of increased knowledge and improved practical capacity. The ordinary doctor, the "mere general practitioner" as he is called, is rapidly becoming in all parts of our own country and the civilised world a man of sound professional culture and of competent bedside resource. This is the kind of fact which the true medical philosopher, and with him every sensible man in the world, perceives to be of the most fundamental and world-wide importance.

### New Drugs and New Names.

Of course, there have been new discoveries, so-called, during the year; and some substances which were not new have received new names—a fact which the pedant will not fail to rejoice over. Creosotal, for instance, may stand as a sample of the new remedies for phthisis. Its name is not yet known to the profession at large, and probably never will be. So far it has worked no miracles. Similarly among new hypnotics Hypnal and Chloralamine; and among anæsthetics and analgesics, Tropococaine may be mentioned. Influenza has also its new and "certain cures"—at least their expounders say so—and among these asaprol may be named as a sufficient example. Of new names for old or modern remedies a few specimens cannot fail to be interesting, alike to "Spooks" and men. Here they are: Phenyldunethylpqrazoline, Salicylbromanilide. Paramonobromophenylacetamide, Tribromphenol, Phenyldehydroquinazoline-hyrdrochloride, Sodiumthioparatoluidinesulphonate, Diethylsulphondmethylethane, Most tremendous and awe-inspiring are these magniloquent orthographical thunders! They keep even the very stupidest of us in countenance as proving that the most sacred and exalted recesses even of the chemical laboratory cannot prevent a fool from being a fool, or from showing himself to be so, to a world which is anything but admiring.

### Reading and Stupidity.

The year has, on the whole, been a good year. Medical men have settled themselves down to solid and earnest work; and medical newspapers and magazines have vied with each other in keeping the whole profession thoroughly instructed and "up to date." In this respect, perhaps, more has been attempted than has been successfully performed. Too much reading tends to mental confusion. Was it Hobbes, the philosopher, who declared that "if he had read as much as the other literary men of his time he would have been as stupid as they were?" We ourselves have made a practical protest against this excessive cramming of the mental stomach with innumerable and often conflicting theories of treatment, by endeavouring week by week to bring the actual hospital ward and its doings into the consulting-room of every medical man in the kingdom. This practical exhibition of actual ward work in its simplest outlines has, we have reason to believe, been much valued by men of all ranks whose business lies at the bedside of the nation. "Practice" is our motto, as it must be the motto of every man who earns his bread, and lives in the public confidence, by ministering to the necessities of the sick. Practice based on science, practice based on experience, practice based on study and on perfect sympathy is, and must be, the Alpha and Omega of every physician or surgeon in the world who is worthy of his honourable name.

